# *qSE7* is a major quantitative trait locus (QTL) influencing stigma exsertion rate in rice (*Oryza sativa* L.)

**DOI:** 10.1038/s41598-018-32629-2

**Published:** 2018-09-28

**Authors:** Keqin Zhang, Yingxin Zhang, Weixun Wu, Xiaodeng Zhan, Galal Bakr Anis, Md Habibur Rahman, Yongbo Hong, Aamir Riaz, Aike Zhu, Yongrun Cao, Lianping Sun, Zhengfu Yang, Qinqin Yang, Liyong Cao, Shihua Cheng

**Affiliations:** 1 College of Agronomy, Shenyang Agricultural University, Shenyang, Liaoning 110866 China; 2 Key Laboratory for Zhejiang Super Rice Research/National Center for Rice Improvement/China National Rice Research Institute, Hangzhou, Zhejiang, 310006 China; 3 Rice Research and Training Center, Field crops Research Institute, Agriculture Research Center, Sakha, Kafrelsheikh, 33717 Egypt; 4 Department of Agricultural Extension, Ministry of Agriculture, Dhaka, Bangladesh

## Abstract

Stigma exsertion is a key determinant to increase the efficiency of commercial hybrid rice seed production. The major quantitative trait locus (QTL) *qSE7* for stigma exsertion rate was previously detected on the chromosome 7 using 75 Chromosome Segment Substitution Lines (CSSLs) derived from a cross between the high stigma exsertion *indica* maintainer XieqingzaoB (XQZB) and low stigma exsertion *indica* restorer Zhonghui9308 (ZH9308). The C51 line, a CSSL population with an introgression from XQZB, was backcrossed with ZH9308 to produce the secondary F_2_ (BC_5_F_2_) and F_2:3_ (BC_5_F_2:3_) populations. As a result, the Near Isogenic Line (NIL *qSE7*^*XB*^) was developed. Analysis indicated *qSE7* acted as a single Mendelian factor and decreased the stigma exsertion. We hypothesized *qSE7* regulates single, dual, and total stigma exsertion rate, provided experimental support. *qSE7* was mapped and localized between RM5436 and RM5499 markers, within a physical distance of 1000-kb. With use of new insertion-deletion (InDel) markers and analysis of the heterozygous and phenotypic data, it was ultimately dissected to a 322.9-kb region between InDel SER4-1 and RM5436. The results are useful for additional identification and isolation of this candidate gene controlling stigma exsertion rate, and provide a basis for further fine mapping, gene cloning, and Marker Assisted Selection (MAS) breeding later.

## Introduction

Rice (*Oryza sativa* L.) is an important staple cereal crop and is a primary source of food for more than half of the global population^1^. It grows worldwide under a wide range of agro-climatic conditions, and thus has wide genetic variety and adaptability. Improvement of hybrid rice breeding could help to address a food shortage problem that is caused by a marked increase in the global population. Stigma exsertion is a major reproductive factor that can increase the opportunity for outcrossing pollination and thus increase yield^2,3^. Stigma exsertion is easily affected by many environmental conditions (wind, temperature, humidity, physical interruption, *etc*.) during the flowering period in rice, and is thus difficult to study^4^.

Exserted stigmas remain viable for about 6 days, with a decrease of 20% in the seed set from cross-pollination per day^5,6^. Maternal parents with a high percentage of exserted stigmas are expected not only to catch more pollen from paternal parents but also to overcome the barriers of flowering synchronization between maternal and paternal parents^7^. Stigma exsertion, including that of single and dual stigmas, together with other floral traits, plays an important role in hybrid seed production, and therefore receives consistent attention from rice breeders and researchers^8–11^. Many quantitative trait loci (QTLs) for stigma exsertion rate have been identified and are distributed across all 12 chromosomes in rice^12–14^.

In hybrid rice breeding programs, the parental generation is used to improve phenotypic traits that increase the natural out-crossing rate, while the cultivated rice is strictly a self-pollinated plant and has floral traits that prevent cross-pollination. Female and pollen parents used for hybrid seed production differ in their desirable traits. Hybrid rice breeders select pollen parents which show traits associated with high yield, whereas the female parents require the trait of male sterility. This has been achieved in rice using cytoplasmic male sterility^15^, which results from mutations in mitochondria, or by genetic male sterility, which occurs from nuclear mutations and includes photoperiod-sensitive and thermo-sensitive genetic male sterility^16^. Although breeders must consider several phenotypic traits that influence the efficiency of hybrid seed production, the frequency of stigma exsertion is most important among them^3^. Several independent studies based on inter- and intra-specific crosses in rice have previously identified quantitative trait loci (QTLs) associated with floral traits. Nine QTLs for the frequency of stigma exsertion have been identified on rice chromosomes 3, 4, 6, 8, 11 and 12^17^. By using the recombinant inbred lines (RILs) derived from a cross between the *indica* cultivar, pei-kuh, and the wild accession W1944 (*Oryza rufipogon*), two QTLs for stigma exsertion were also found on chromosomes 5 and 10^18^.

Rahman *et al*.^19^ conducted an experiment for QTL mapping of the stigma exsertion rate and spikelet number per panicle in rice (*Oryza sativa* L.) using 134 RILs derived from a cross between the parents XQZB and ZH9308. This study detected eight QTLs for stigma exsertion on chromosomes 1, 6, 10, and 11. They fine mapped the new QTL *qSE11* to a narrow distance nearly about 350-kb, with genetic dissection and validation^20^. Through phenotypic and genotypic experiments, QTLs controlling stigma exsertion rate were revealed to be clustered in two intervals on chromosome 7^4^. A different study detected another QTL cluster affecting stigma exsertion rate on chromosome 7 in rice^21,22^.

**Figure 1:**
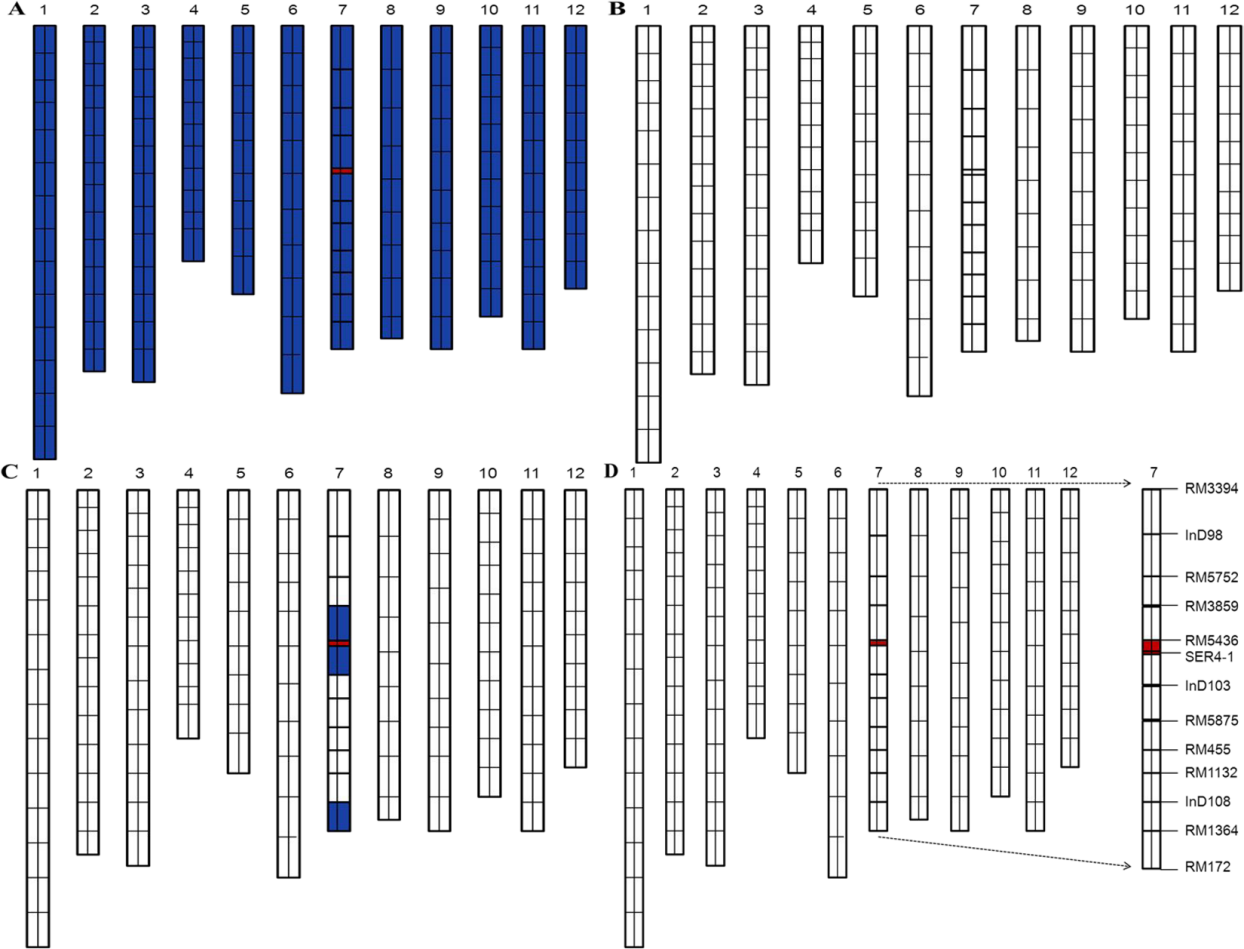
The rice chromosomal map. (**A**) The genetic map of XQZB; (**B**) the genetic map of ZH9308; (**C**) the genetic map of C51; (**D**) the genetic map of NIL (*qSE7*^XB^). blue regions indicate the genotypes of the homozygous XQZB allele; White regions indicate the genetic background of ZH9308; red regions indicate the major QTL (*qSE7*) allele.

In this study, we used the C51 line, a CSSL containing one introgression segment from the donor parent (XQZB) on chromosome 7 that increases the stigma exsertion rate. Three flowering related traits, including SSE (single stigma exsertion), DSE (dual stigma exsertion), and TSE (total stigma exsertion) were measured for mapping of primary QTLs. C51 is derived from the parental populations XQZB and ZH9308 (Fig. 1), which have high and low stigma exsertion rates, respectively. We dissected the major QTL (*qSE7*) and delimited it into a narrow region on chromosome 7, and hypothesized that *qSE7* controls both SSE and DSE.

## Results

### Construction of NIL (*qSE7*^*XB*^)

**Figure 2:**
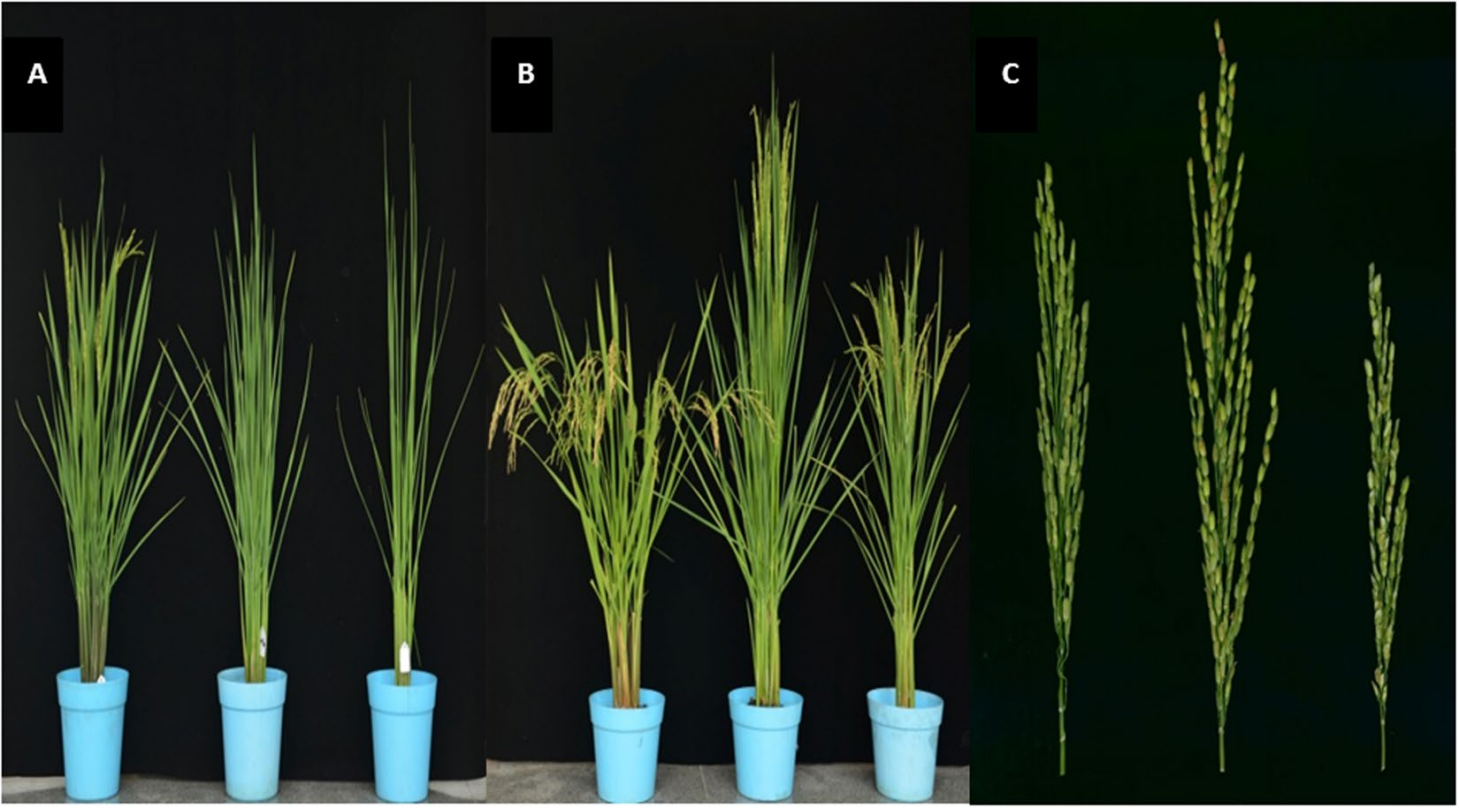
The phenotype of the parents XQZB (left in each panel), ZH9308 (middle), and NIL(*qSE7*^*XB*^) (right); (**A**,**B**) show the whole plants, (**C**) shows only the panicles of the plants; (**A**–**C**) represent 65, 91 and 80 days after transplanting, respectively. The genotypes of XQZB, ZH9308 and NIL (*qSE7*^XB^) are shown in (**A**–**C**) of Fig. 1, respectively.

**Figure 3:**
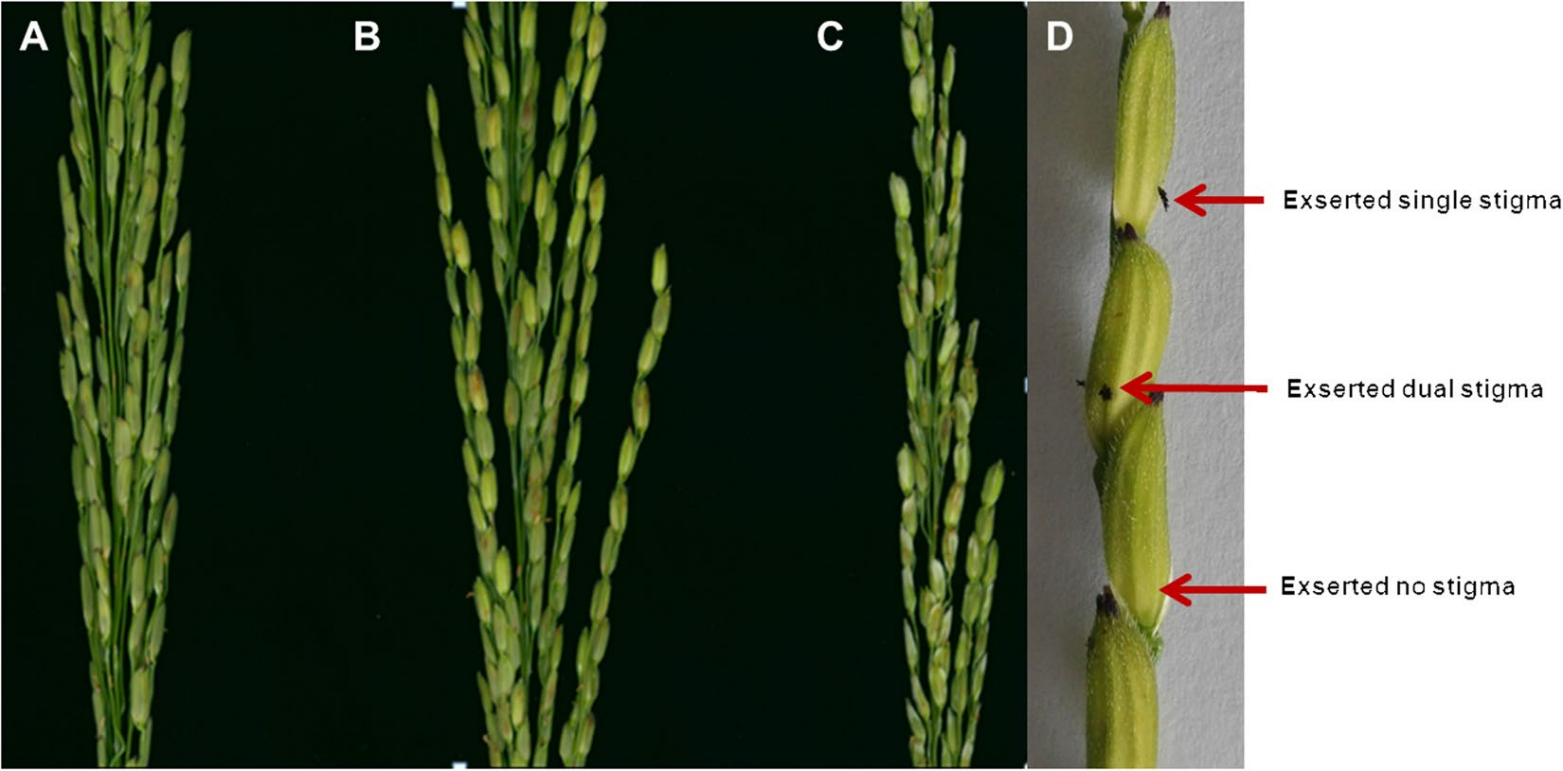
Phenotypes of the exserted stigma of the parents. (**A**) XQZB; (**B**) ZH9308 (stigma are brown and not clearly visible); (**C**) NIL (*qSE7*^*XB*^); (**D**) examples of single, dual exserted stigmas, and of no exsertion, in a spikelet in this study. The genotypes of XQZB, ZH9308 and NIL (*qSE7*^XB^) are shown in (**A**–**C**) of Fig. 1, respectively.

Using the CSSL populations, the primary QTL *qSE7* was detected in the interval between SSR markers RM5436 and RM5875 of chromosome 7 and was validated in the secondary F_2_ population derived from the cross between C51 and ZH9308 (unpublished data). The C51 line of the BC_4_F_9_ population only showed homozygous XQZB alleles in the introgressive interval and had uniform stigma exsertion, and was thus selected for backcrossing with the recurrent parent ZH9308 to develop the secondary F_2_ and F_2:3_ populations. We used 120 genome-wide SSR and InDel markers^23^, which were distributed along the rice genome, to compare to previously reported linkage maps^19^. The C51 and secondary F_2_ populations were used for validation and narrowing of the major QTL *qSE7*, while the F_2:3_ population was used for genetic validation and dissection. C51 was homozygous for XQZB alleles in the targeted QTL region on chromosome 7, while more than 90% of genetic background was from ZH9308. Several chromosome fragments across chromosome 7 were heterozygous in the secondary F_2_ population. Finally we selected the line with the least background donor introgression, with only three small heterozygous regions in the area of chromosome 7 which contained the targeted QTL (*qSE7*). The rice chromosomal map is shown in Fig. 1. Based on the phenotypic performance (Figs 2 and 3), QTL validation, and the genome-wide selection, we constructed one NIL (*qSE7*^*XB*^), which has homozygous XQZB regions surrounding the *qSE7* allele.

### Percentage of Stigma Exsertion in XQZB, ZH9308, C51, and NIL (*qSE7*^*XB*^)

Phenotypic data showed that the values of SSE, DSE, and TSE varied significantly across the XQZB, ZH9308, C51, and NIL (*qSE7*^*XB*^) populations (Table 1, Fig. 2). However, these values did not differ detectably between C51 and NIL (*qSE7*^*XB*^) according to the Student’s *t*-test. In the secondary F_2_ segregating population, the mean values of the SSE, DSE, and TSE were 22.92, 3.09, and 26.01%, respectively, while the peak values were 36.12, 7.62, and 40.93%.

**Table 1:** Stigma exsertion rates for XQZB, ZH9308, C51, NIL (*qSE7*^XB^), and the F2 population derived from C51 and ZH9308.

Traits	XQZB ± SE	ZH9308 ± SE	C51	NIL(qSE7XB)	F2 population developed from CL51 and ZH9308	
					Mean	Range
SSE	36.49 ± 0.92^a^	11.69 ± 0.73^b^	6.41 ± 0.52^c^	3.35 ± 0.42^d^	22.92 ± 0.03	7.10–36.12
DSE	7.56 ± 0.81^a^	1.17 ± 0.62^b^	0.72 ± 0.72^c^	0.49 ± 0.66^d^	3.09 ± 0.02	0.14–7.62
TSE	44.04 ± 0.96^a^	12.86 ± 1.10^b^	7.14 ± 1.00^c^	3.84 ± 0.81^d^	26.01 ± 0.05	8.31–40.93

SSE, DSE, and TSE indicate the single stigma exsertion, dual stigma exsertion and total stigma exsertion rates, respectively; Superscript letters indicate statistically significant (p < 0.01; student’s *t*-test) differences between the mean values in each row; NIL(*qSE7*^XB^) is the studied population carrying the homozygous *qSE7* locus from XQZB with the primary genetic background of ZH9308; ± SE, standard error.

**Figure 4:**
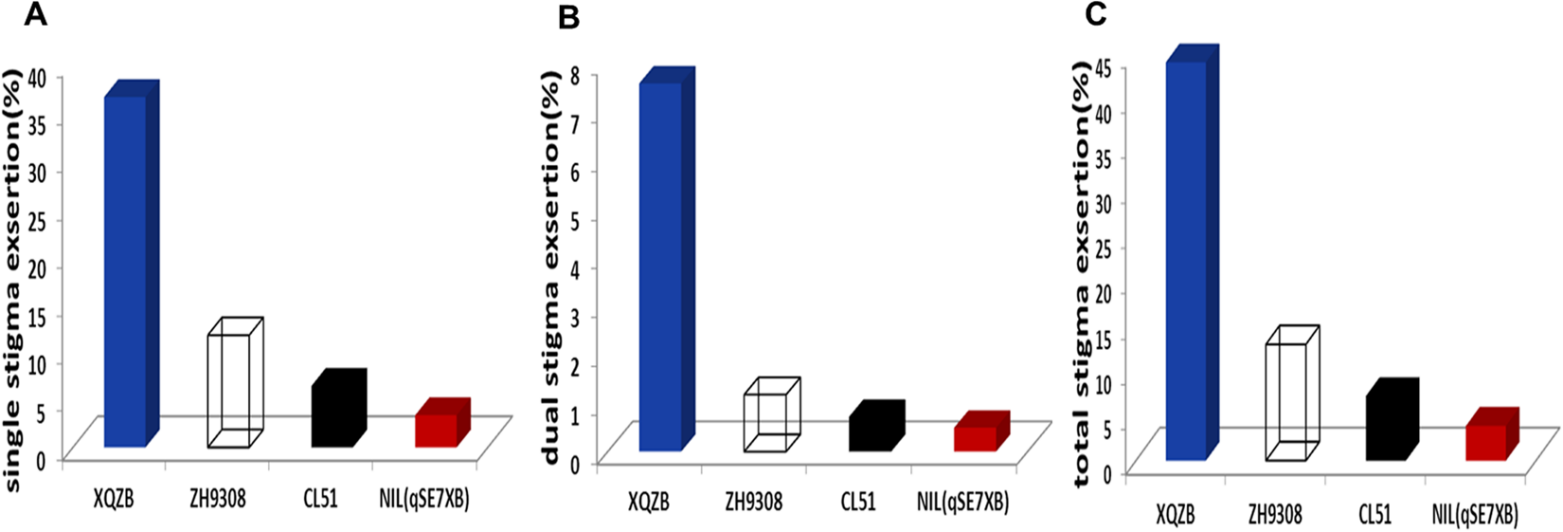
Comparison of stigma exsertion rates during flowering periods in the field for QTL validation. (**A**) Single stigma exsertion (SSE); (**B**) dual stigma exsertion (DSE); (**C**) total stigma exsertion (TSE).

In our study, a highly significant QTL (*qSE7*) was predicted in the NIL (*qSE7*^*XB*^), and its location was validated on the long arm of chromosome 7. The genotypes of the *qSE7* locus for the F_2_ plants were easily detected by measuring the stigma exsertion rate of their progeny. The SSE, DSE, and TSE of the recurrent parent ZH9308 were 11.69, 1.17, and 12.86%, respectively, while those of the NIL phenotype were 3.35, 0.49, and 3.84%. Compared to the recurrent parent ZH9308, the NIL showed stigma exsertion reduced 8.3, 0.7 and 9.0% for SSE, DSE, and TSE, respectively (Fig. 4).

### Phenotypic analysis of the stigma exsertion rate in the secondary F_2_ population

**Figure 5:**
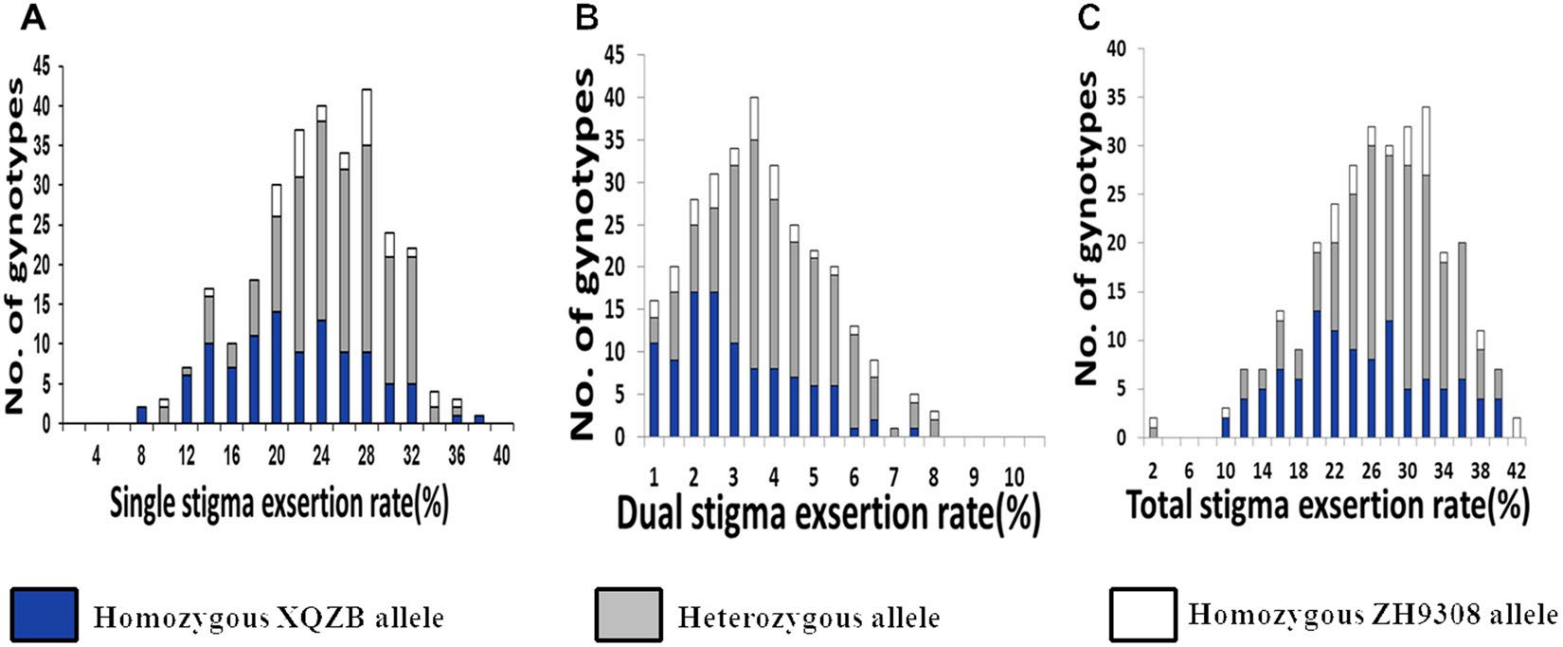
Frequency distribution of stigma exsertion rates in the secondary F_2_ population. (**A**) Single stigma exsertion (SSE); (**B**) dual stigma exsertion (DSE); (**C**) total stigma exsertion (TSE); blue regions indicate the genotypes of the homozygous XQZB allele; gray regions indicate the genotype of the heterozygous allele; white regions represent the genotype of the homozygous ZH9308 allele.

The three phenotypic traits of SSE, DSE, and TSE were measured in 4000 secondary F_2_ individuals. These traits frequencies were distributed continuously and followed a normal distribution (Fig. 5). Gene frequencies were distributed according to the Mendelian ratio of 1:2:1 (X^2^ = 0.25 < X^2^_0.05_ = 3.19), indicating that the QTL was likely controlled by one genetic locus. Expression of the *qSE7* QTL was relatively stable in the secondary F_2_ population, where it had a substantial effect on stigma exsertion rates.

The correlation coefficients among the three traits (SSE, DSE, TSE) are shown in Table 2. There was significant correlation among these three traits in the secondary F_2_ segregating population. SSE and TSE showed the highest phenotypic correlation (r = 0.988**), followed by DSE and TSE (r = 0.808**), while SSE and DSE have the lowest correlation (r = 0.710**). These results demonstrate that the lines which had higher single stigma exsertion rate were more likely to also show increased total stigma exsertion rate as well as dual stigma exsertion rate.

**Table 2:** Correlation (Pearson) coefficients among the SSE, DSE, and TSE traits in the F_2_ population.

	SSE	DSE	TSE
SSE	1		
DSE	0.710**		
TSE	0.988**	0.808**	

**Correlation was considered significant at the 0.01 level (2-tailed); SSE, DSE, and TSE represent single stigma exsertion, dual stigma exsertion, and total stigma exsertion rates, respectively.

### Primary mapping of *qSE7* the QTL

The major QTL (*qSE7*) on chromosome 7 responsible for stigma exsertion rate was detected with LOD values ranging from 1.76 to 3.91, and the phenotypic variance controlled by each QTL ranged from 5.15 to 17.20% (Table 3).

**Table 3:** Major quantitative trait loci (QTLs) for stigma exsertion rates detected in the CSSL and the secondary F_2_ populations derived from the parental lines XQZB and ZH9308.

Trait	QTL	Chr	Markers	Sources	CSSLs(75Lines)		A (%)	BC6F2	PVE (%)	A (%)
					LOD	PVE (%)		LOD		
SSE	qSSE7	7	RM5436-RM5499	ZH9308	2.01	5.93	1.52	2.92	15.16	8.81
DSE	qDSE7	7	RM5436-RM5499	ZH9308	1.76	5.15	1.02	3.91	17.20	12.6
TSE	qTSE7	7	RM5436-RM5499	ZH9308	2.03	6.01	1.41	2.82	15.31	8.01

*qSSE*, *qDSE*, and *qTSE* represent the QTL for the single stigma exsertion rate, dual stigma exsertion rate, and total stigma exsertion rate, respectively; A, the additive effect of each QTL; PVE, the phenotypic variance explained by each QTL; LOD, logarithm of odds.

**Figure 6:**
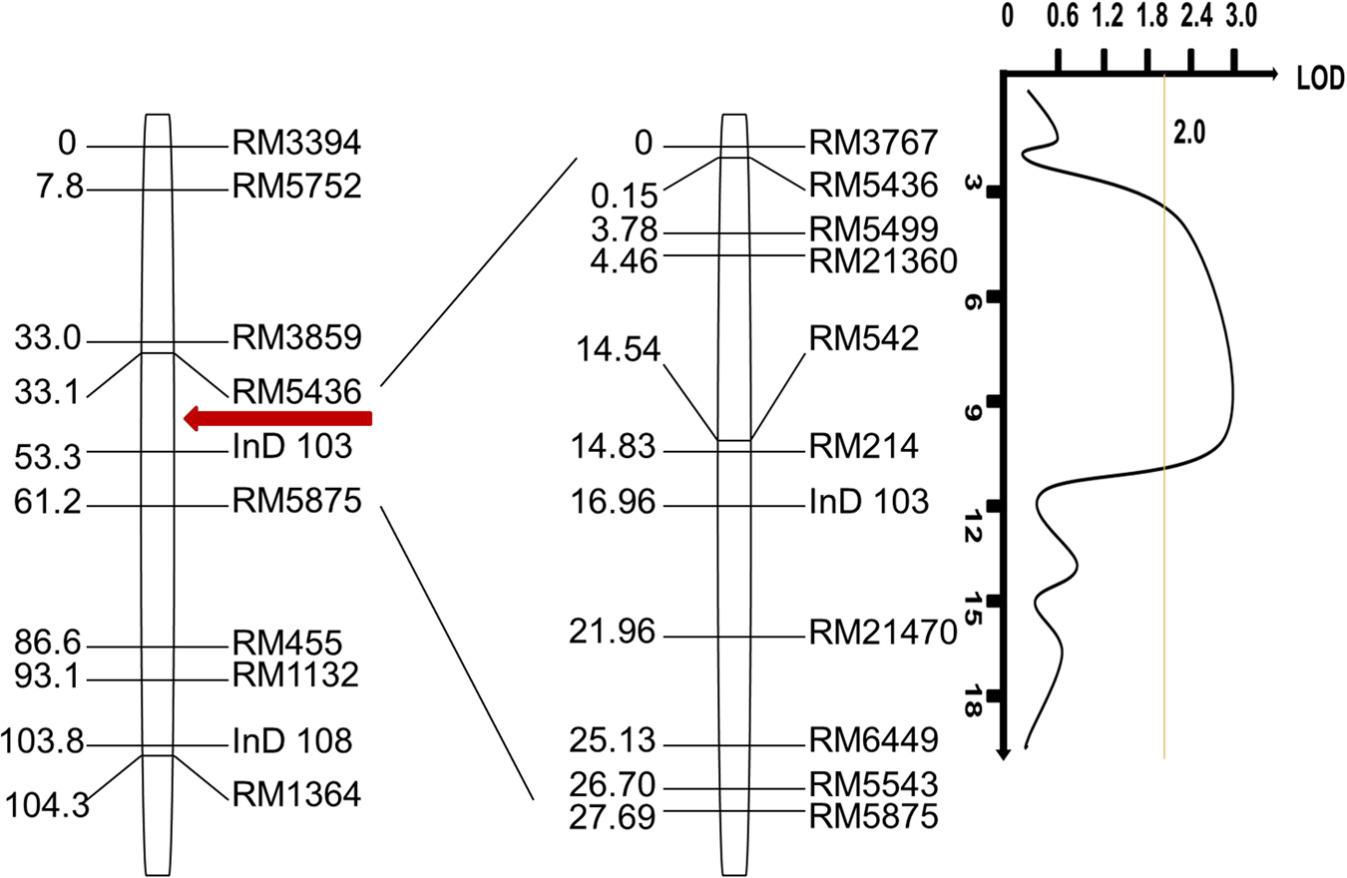
Genetic linkage analysis of the *qSE7* allele on chromosome 7; red arrow indicates the location of *qSE7*.

The results of a Chi-squared test of the phenotypic and genotypic values for the 300 randomly selected samples showed that the separation of the SSE fit a Mendelian single factor ratio of 3:1 (Fig. 6).

**Figure 7:**
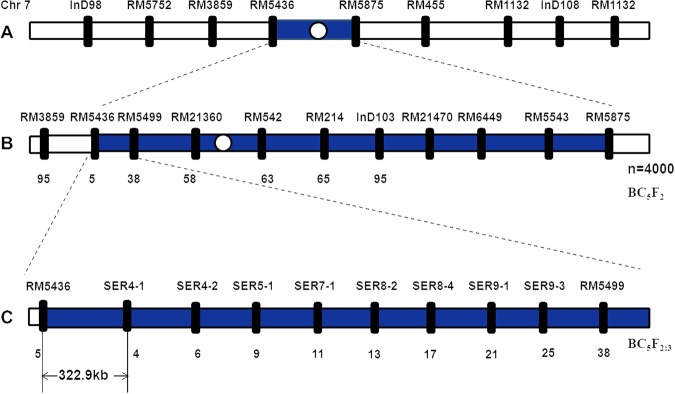
Mapping and dissection of the *qSE7* on chromosomal and physical maps. (**A**) *qSE7* was primarily mapped to the region between RM5436 and RM5875 on chromosome 7. (**B**) Validation of the *qSE7* based on the secondary F_2_ populations; the locus was mapped to the region between RM5436 and RM5499; numbers below blue line indicate heterozygous individuals identified in the secondary F_2_ population. (**C**) Genetic dissection of the *qSE7* based on the F_2:3_ populations; the allele was mapped to the physical location of 322.9-kb in the region between the markers RM5436 and SER4-1.

Using the markers RM5436 and RM5875 to analyze 4000 plants of the F_2_ population derived from C51 and ZH9308, we found 95 heterozygous seeds. When we used the 8 newly developed SSR markers for the same analysis, we found fewer and fewer heterozygous seeds, such as 65, 63, 58, 38 and 5 (Fig. 7).

To develop the new InDel markers, we ordered 30 InDel Primers and only 8 of these primers showed polymorphism between the two parents of the C51 population (Table 4). When using these newly developed InDel Markers, we found no changes across the 38 heterozygous seeds (by using the SSR marker RM5499). Thus, the physical distance of the QTL containing *qSE7* is nearly 1000-kb, located between RM5436 and RM 5499.

**Table 4:** Sequences of new SSR and InDel markers designed for a study of QTLs for stigma exsertion rate in rice.

Primer name	Forward primer(5′-3′)	Reverse primer(5′-3′)	Production length/bp	Purpose
RM5436	TGAGCTGCACAAGACAGACAAGC	ACCATTTGAACAGGATGGACTGG	150	primary mapping
RM5875	AATAAAGCGAGATGGACGAACC	TTTCCCACCAGAGGAAGATGG	90	primary mapping
InD 103	CCCCATGAGGCCTACACTT	AGCAGCATAATCAGATGAGACG	100	primary mapping
SER4-1	CTGGTGAATTCGACATGTGCC	GAGTGGGTGGCTGCTACTG	200	Dissection
SER4-2	CGAAAAGAGTTTTGCCCTTTTGC	CAAGGAAAGGCTGCACAACAG	130	Dissection
SER5-1	AGGATGGATCCGATACTTTTAGCTT	CTGGCTCCTAATAGTACTGCTGA	180	Dissection
SER7-1	TCTCAACAGGGCCTCTCCAA	CGAGACTGAAGTCAGACCAGT	120	Dissection
SER8-2	GATGTACCCGAGTCTTCTGAAAT	AAAGCAGTGGCGAGCAGATT	105	Dissection
SER8-4	ACGGTGTATAAATAGTTTCATCGAG	TTGAACCTGCCGACGTCTC	185	Dissection
SER9-1	ACACCATTCTTTCCAGCCAAC	AGAACATGGAAGCCTTATTCAACT	150	Dissection
SER9-3	TGGGCTAAGGGAATTTGCGA	TGGTTTCTTCGTAGTAACGCATC	140	Dissection

With genetic and phenotypic data analysis, we validated the QTL (*qSE7*) to a region of 1000-kb. To narrow the allele to a smaller region, we developed 8 new InDel markers in the interval between RM5436 and RM5499 (Fig. 7A,B). With high-resolution genetic dissection analysis of 3200 individuals from F_2:3_ population using these newly developed markers, we detected fewer heterozygous seeds. The location of the *qSE7* was narrowed to a region of 322.9-kb between the RM5436 and SER4-1 markers (Fig. 7C).

### Further mapping and dissection of the *qSE7* QTL

**Figure 8:**
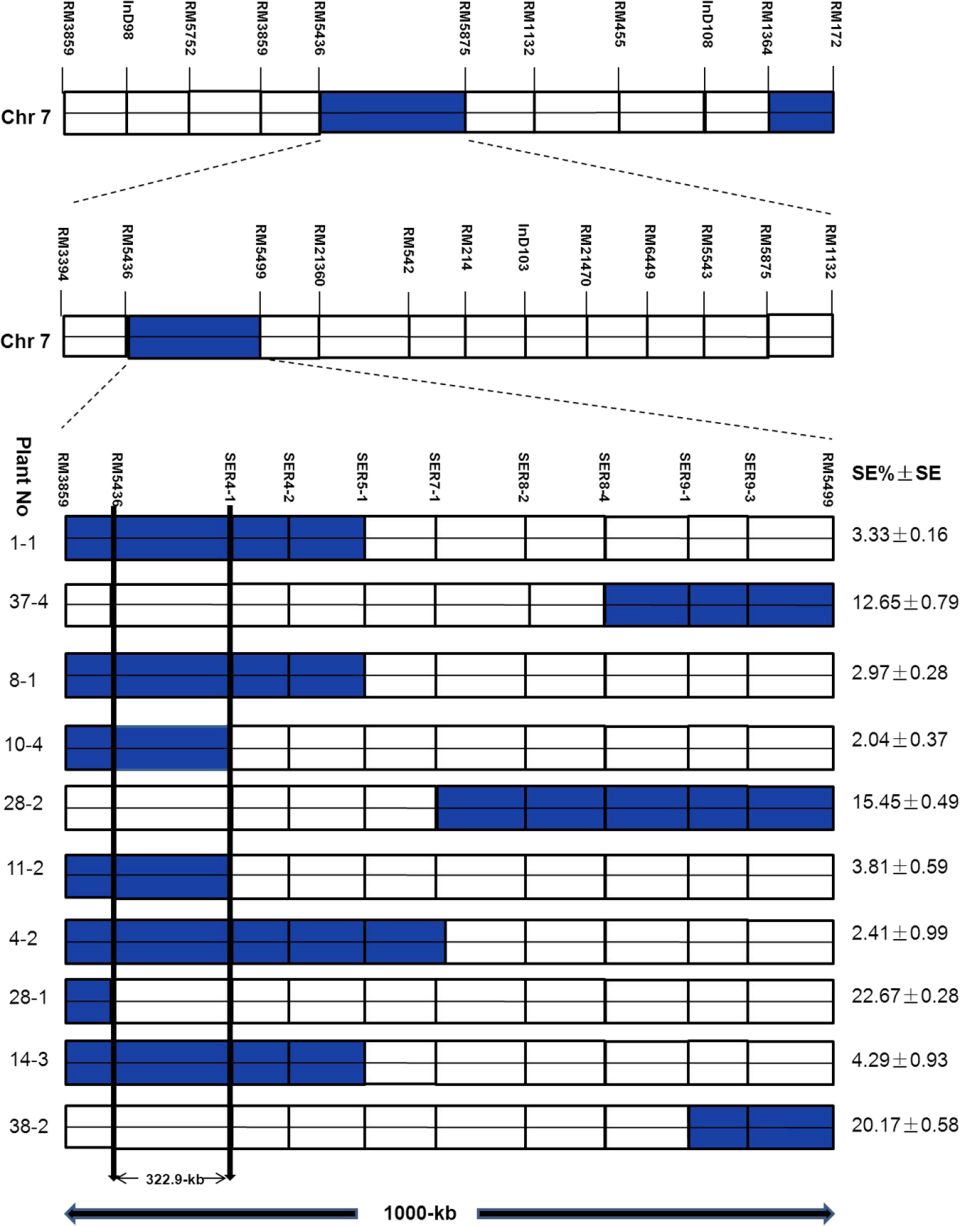
Genetic anatomy of homozygous recombinant plants in the F_2:3_ populations for a region of 1000-kb between the RM 5436 and RM5499 markers on chromosome 7, with the position of 11 newly developed markers. White regions indicate ZH9308 genetic background; blue regions indicate XQZB locus introgression; the segment between RM 5436 and SER4-1 is designated the major QTL(*qSE7*) allele; SE (%): Stigma exsertion rates; SE: Standard error.

By using the phenotypic and genotypic data of the F_2:3_ population, we further mapped the QTL to a narrow range of about 322.9-kb. A chromosomal dissection showed that each homozygous recombinant plant contained at least one donors (XQZB) introgression segments, as well as recipient (ZH9308) segments and the major QTL (*qSE7*) allele (which was located between RM5436 and SER4-1) on chromosome 7 (Fig. 8). The length of substituted chromosome segments in homozygous plants was detected according to the response of the markers. Each chromosomal segment was estimated between two markers.

By analyzing the genetic and phenotypic data, we validated the phenotypic performance of the stigma exsertion rate, ranged from 2.04–22.67% (Fig. 6) in these homozygous recombinant plants, the targeted region containing *qSE7* was finally narrowed to a region of 322.9-kb between RM5436 and SER4-1.

## Discussion

Food security has become a global problem. One approach to solving this problem is increasing productivity of the major crop plants^24^. A higher stigma exsertion rate can promote the seed production of hybrid rice. For this reason, research has begun to focus on the genetic basis for stigma exsertion rate.

Numerous studies have described QTLs associated with the stigma exsertion traits of SSE, DSE, and TSE in rice. For example, 5 QTLs were found to be distributed on chromosomes 2 and 3^25^; 9 QTLs were found on chromosomes 1, 2, 5, and 8^26^; 15 QTLs were discovered on chromosomes 1, 5, 6, 7, 8, 9, 10, and 11^4^; 6 were found on chromosomes 1, 2, 5, and 8^27^; 5 were on chromosomes 5, 6, and 7^21^. Three QTLs were detected on chromosome 7 that affected stigma L/W (Length/Width) ratio, dual stigma exertion rate, and total stigma exertion rate^4^. The QTL for stigma L/W ratio is linked to marker RM118, and the QTLs for PDSE and PTSE are linked to marker RM455. These are located at the top of chromosome 7, a significant distance from the location of the locus *qSE7* which influenced stigma exertion rate in our study. Li *et al*.^22^ found 11 QTLs on chromosomes 1, 3, 6, 7, 9, 10, and 12. Among these, the QTL on chromosome 7, *qSSE7*, was located near the QTL found by Yan *et al*.^4^ at the bottom of the long arm of chromosome 7. Another QTL on chromosome 7 is linked with RM650 and is distant from the *qSE7* described in this study^21^, so to the best of our knowledge, *qSE7* is a previously-undescribed gene.

In a previous study, we identified one QTL for the stigma exertion rate on chromosome 7. By using the C51 population, we identified the locus on chromosome 7 containing this QTL as *qSE7*. Furthermore, we dissected this new gene region containing *qSE7* to a narrow interval of about 322.9-kb. One QTL for stigma exertion rate was identified in the interval between RM5436 and RM5875 on chromosome 7 in our previous study, which used 75 CSSLs to identify the primary QTLs. In this study, we narrowed the segment containing the locus *qSE7* to a small interval of approximately 1000-kb between RM5436 and RM5499 by using 300 F_2_ individual plants. Furthermore, the genotypic and phenotypic data from of 3200 F_2:3_ plants were used to map this locus, and we narrowed this to a small interval of 322.9-kb between RM5436 and SER4-1.

We have constructed a set of genome-wide CSSLs, consisting of 75 lines which carry genomic introgression fragments from the donor parent of XQZB in the genetic background of ZH9308. These populations are now used to analyze the genetic basis of complex traits such as TGWT (1000 grain weight), GN (Grain Number), and the stigma exsertion traits of SSE, DSE, and TSE. In our study, we used C51, one of the CSSLs containing introgressive segments on the long arm of chromosome 7, to clarify the stigma exsertion rate. A major QTL (*qSE7*) for stigma exsertion rate was identified as a single Mendelian factor. The most significant effect of the *qSE7* allele was decreasing the stigma exsertion rate in rice. A significant positive correlation was observed among SSE, DSE, and TSE. The gene *qSE7* also controls the SSE, DSE, and TSE, and can decrease the stigma exsertion rate.

We identified the major QTL (*qSE7*) on the long arm of rice chromosome 7 by using the chromosome segment substitute line C51. This CSSL inbred line with more than 90% of the genetic background of ZH9308 was selected to produce NIL (*qSE7*^*XB*^) to target the region of interest. In the secondary F_2_ population, the frequency distribution of the stigma exsertion rate was shown to be discontinuous. This F_2_ population was used to validate the QTL, and we found that the ranges of phenotypic variation of SSE, DSE, and TSE were 7.10 to 36.12, 0.14 to 7.62, and 8.31 to 40.93%, respectively. The mean values of SSE, DSE, and TSE in the F_2_ population were 22.92, 3.09, and 26.01%, respectively (Table 1). 3200 individuals of F_2:3_ were further used to dissect the *qSE7* region. The SSE, DSE, and TSE of the recipient parent ZH9308 line were 11.69, 1.17, and 12.86%, respectively, while the SSE, DSE, and TSE of the NIL were 3.35, 0.49 and 3.84% (Table 1). The action of the targeted allele *qSE7* decreased the stigma exsertion rate by 8.3, 0.7 and 9.0% for SSE, DSE, and TSE, respectively, compared to values for the recurrent parent ZH9308 (Table 1). This result indicated that *qSE7* is responsible for the low stigma exsertion rate in the NIL population.

The *qSE7* generated from XQZB had a negative effect, decreasing the SSE, DSE, and TSE. Although the SSE of XQZB was higher than that of ZH9308, the final phenotype results from not only one gene, but all genes and their interactions across the 12 chromosomes in XQZB. The *qSE7* gene region has a negative effect although the overall effect of these genes on XQZB is positive. Thus, the allele containing the QTL (*qSE7*) derived from XQZB can decrease the stigma exsertion rate in rice. This suggests that *qSE7* has a negative effect and is responsible for the low stigma exsertion rate in the NIL, and that *qSE7* from ZH9308 is a promising QTL for the development of a high stigma exsertion maternal line for hybrid rice seed production. Rice breeders should therefore use MAS to select against the *qSE7* region of chromosome 7 in XQZB, and thereby develop a new CMS (Cytoplasmic Male Sterility) with high stigma exsertion to improve the potential products of hybrid seeds in breeding practice.

The secondary F_2_ population was heterozygous in the *qSE7* region and fixed at the *qSSE7*, *qDSE7*, and *qTSE7* loci. *qSE7* increased the variation in stigma exsertion in the F_2_ population, so its progeny showed very wide variation. By using the newly developed molecular markers (Table 4) in the targeted region, we evaluated the gene effect on 3200 plants of the F_2:3_ populations. Homozygous recombinant plants were selected and the phenotypic performance of the SSE, DSE, and TSE was validated. 8 newly developed InDel markers were used for dissection of *qSE7*. In the secondary F_2_ population, the frequency distribution of the stigma exsertion rate followed the expected Mendelian ratios (1:2:1) for single locus segregation, and was shown to be discontinuous, so *qSE7* was mapped to a 1000-kb region between the two markers RM5436 and RM5499. With the phenotypic data collected from the F_2:3_ population, the gene *qSE7* was finally narrowed down to a region of 322.9-kb, and 8 newly developed sequence tagged markers were used for high resolution mapping.

To dissect the genetic basis of important stigma exsertion traits, many segregating populations were used for primary mapping, including the F_2_ population, RILs, and DHs (Doubling Haploids). Advanced populations such as NILs and CSSLs, are practical tools for genetic mapping and cloning^28–33^. These populations were composed of several lines containing a single fragment, or a small number of introgressive fragments, from a donor parent into another with a homogeneous genetic background. Because of the minimized genetic background noise, targeted QTL can be considered as a single Mendelian factor and thus be researched and isolated.

QTLs with large effects have been identified as a valuable resource for the genetic improvement of quantitative traits^34,35^. As global population increases in tandem with a changing climate, increased production of sustainable rice is required^36^. Marker-assisted selection (MAS) has become popular in recent years due to its ability to reduce the cost and improving the efficiency and accuracy of seed selection^37,38^. For successful MAS application, DNA markers tightly linked to a targeted trait must be identified and can be used as a band for screening phenotypic variances. Improvement of the use of MAS requires a better understanding of the genetic basis of all focused traits, development of molecular markers linked to targeted genes, and study of the allelic variation at those loci. Although MAS is rapidly improving, few reports have been made on its use in quantitative trait improvement, particularly for the stigma exsertion rate in rice. The instability of QTL expression and the lack of reliable markers are currently the main barriers to large-scale utilization of MAS in high-yield hybrid rice breeding.

In this study, we identified and confirmed the major QTL *qSE7* on the long arm of chromosome 7 using the C51, NIL, F_2_, and F_2:3_ populations, and narrowed it to a region of 322.9-kb between RM5436 and SER4-1. This region has never been reported to be involved in stigma exertion rate, so this was considered a new allele. By using the QTL (*qSE7*), we developed a NIL which showed decreased frequency of exserted stigmas by 8.3, 0.7 and 9.0% for SSE, DSE, and TSE, respectively, compared to that of the recurrent parent ZH9308. This result indicated that *qSE7* derived from XQZB is a negative QTL, and selection should be avoided in rice breeding. The gene variant from ZH9308 could be used in the construction of high stigma exserted maternal lines for hybrid rice seed production. Stigma exertion is sufficient for cross pollination in hybrid rice seed production, and this advantage can be used to improve the products of rice hybrid seeds with further genetic enhancement of CMS through MAS in the future. Our work finely mapped *qSE7* to a narrow 322.9-kb region and provided the groundwork for future gene cloning and MAS. Rice breeding programs will benefit from enhancement of stigma exsertion in CMS lines of hybrid rice.

## Methods

### Materials and Field Experiments

**Figure 9:**
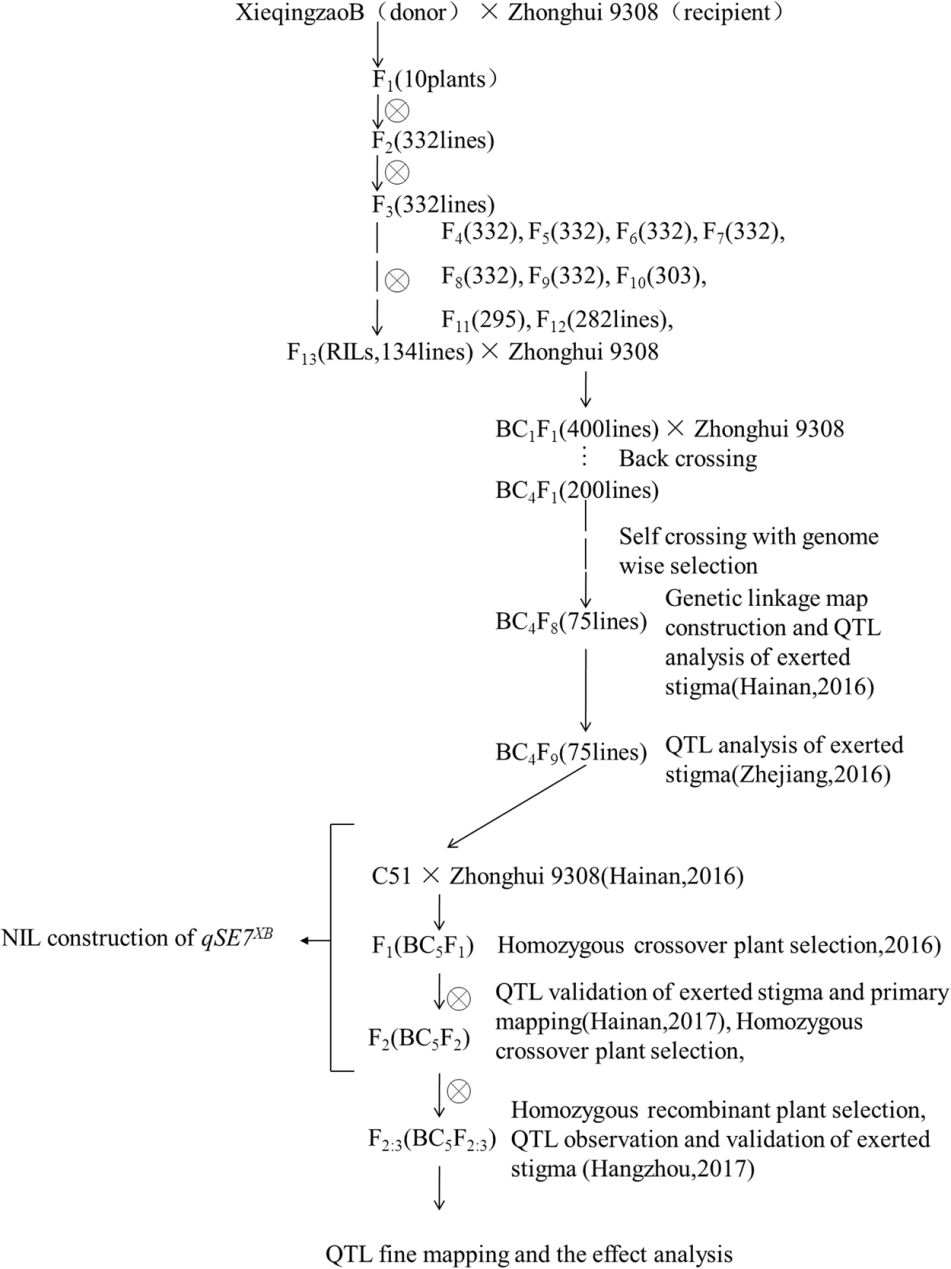
Work flow for material development in a study of QTLs for stigma exsertion rate in rice.

We developed a set of 75 lines of CSSL populations by using XQZB (high stigma exsertion) as the donor parent and ZH9308 (low stigma exsertion) as the recurrent parent. The C51 line is one of these CSSL populations, with an introgression from XQZB and overall genetic background from ZH9308. The C51 line was back-crossed with ZH9308, and the secondary F_2_ and F_2:3_ generations were developed by subsequent selfing in order to dissect and validate the QTLs for stigma exsertion rate. The C51 has both a segment from XQZB and a much lower stigma exsertion rate than the parental populations (Table 1), indicating that the QTL containing *qSE7* has a negative effect on stigma exsertion rate. Thus, we produced the NIL (*qSE7*^*XB*^) with an introgression segment from XQZB, which had a significantly lower stigma exsertion rate than did the parents. A schematic of detailed work flow for the population development is shown in Fig. 9.

The C51 line was crossed with ZH9308 as the recurrent parent and planted in Lingshui, Hainan Island, China on 4 January 2016. The F_1_ plants and their parents were planted in the Fuyang field of the China National Rice Research Institute, Hangzhou, Zhejiang province, China on 15 June 2016. The F_2_ population, the NIL (*qSE7*^*XB*^), and their parents were planted in Lingshui, Hainan Island, China on 6 January 2017. The F_2:3_ populations, the NIL (*qSE7*^*XB*^), and their parents were planted in the Fuyang field of the China National Rice Research Institute, Hangzhou, Zhejiang province, China on 15 June 2017. Each of the progenies were established in 6 rows with 8 plants per row, with spaces of 30 and 20 cm within and between rows, respectively. Rice was grown according to standard cultivation practices.

### Traits Evaluation

Panicles were sampled 6 to 7 d after flowering. We collected 3 normal panicles from each plant in the secondary F_2_ population in Hainan and the F_2:3_ population in Zhejiang, and 5 panicles from each parent and the NIL (*qSE7*^*XB*^). After the lower side spikelets of the panicle flowered, the stigma exsertion rate was observed. The percentage of exserted stigma was calculated by the method proposed by Miyata *et al*. with minor modifications^39^. We measured three traits for percentage of stigma exsertion rate: single stigma exsertion (SSE), dual stigma exsertion (DSE), and total stigma exsertion (TSE). The counts of stigma exsertion were converted by using the following formulae:

$$\begin{array}{rcl}{\rm{SSE}}\,( \% ) & = & [{\rm{SSE}}/({\rm{SSE}}+{\rm{DSE}}+{\rm{Percentage}}\,{\rm{of}}\,{\rm{stigma}}\,{\rm{not}}\,{\rm{exserted}})]\times 100\\ {\rm{DSE}}\,( \% ) & = & [{\rm{DSE}}/({\rm{SSE}}+{\rm{DSE}}+{\rm{Percentage}}\,{\rm{of}}\,{\rm{stigma}}\,{\rm{not}}\,{\rm{exserted}})]\times 100\\ {\rm{TSE}}\,( \% ) & = & {\rm{SSE}}\,( \% )+{\rm{DSE}}\,( \% )\end{array}$$

### DNA Extraction and PCR Products Analysis

Total genomic DNA was extracted from fresh leaves of 4000 plants of the F_2_ population and 3200 each of the 3200 F_2:3_ and parental populations using the Cetyltrimethyl Ammonium Bromide (CTAB) method as described by Luo *et al*.^40^. The extracted DNA was dissolved in Tris and EDTA (TE) buffer and tested for quality and quantity using a DU 640 nucleic acid and protein analyzer (Beckman Coulter Co. Brea, CA, USA). These DNA samples were diluted to 25 ng/μl with sterilized double distilled water and stored at −20 °C for polymerase chain reaction (PCR) amplification, performed according to the methods of Luo *et al*.^40^ in a Thermo Hybrid MBS 0.2S PCR Thermal Cycler (Fisher Scientific International, Hampton, NH, USA). PCR products were separated on 8% non-denatured polyacrylamide gel electrophoresis and detected by silver staining^22^.

### Development of New SSR and InDel Markers

The Simple Sequence Repeat (SSR) database (http://www.gramene.org/microsat) was used to find new SSR markers in the primary QTL interval between RM5436 and RM5875.

Development of new InDel markers was conducted by comparing the sequences of XQZB and ZH9308 to identify areas of difference. We used the online software Primer BLAST (http://www.ncbi.nlm.nih.gov/) to develop new InDel markers in the locus where more than 10 bp insertion or deletion occurred. (The PCR products were about 100–300 bp in length).The primers for these new SSR and InDel markers were obtained through the Tingke Technical Corporation, Hangzhou, China. All new markers designed in this study are shown in Table 4.

### Target Segment Narrowing and Validation

After marking the target interval, with the target gene and heterozygous loci between the newly developed markers, the final molecular markers linked with the goal locus were determined.

From the 4000 sampled plants of the F_2_ population, we randomly selected 300 samples to detect and validate the QTL, and then narrow to its location in the target segment. Each of the 4000 F_2_ plants was used to detect the heterozygous seeds (as shown in Fig. 7). To validate this interval, we obtained 58 new SSR markers in the interval between RM5436 and RM5875. Of the 58 SSR markers, only 8 showed polymorphism between the two parents. We used these 8 SSR markers in 300 random samples to narrow and validate the *qSE7* QTL with phenotypic and genotypic evaluation. We used the software Windows QTL Cartographer V2.5 (http://statgen.ncsu.edu/qtlcart/WQTLCart.html), and a logarithm of odds (LOD) value of 2.0 was used as the standard for the presence of putative main-effect QTLs.

### Further mapping of the allele

RM5436 and RM5499 were used to select the heterozygous plants from the 4000 individual plants of the secondary F_2_ segregating population. We designed 8 new InDel markers to narrow the distance using 3200 individuals of the F_2:3_ populations. In the F_2:3_ population, we used the new markers in the targeted region, evaluated their genetic effect, then selected the homozygous recombinant plants. Finally, we validated the phenotypic performance of the stigma exsertion rate, which ranged from 2.04–22.67% in these homozygous recombinant plants. By analyzing the genetic and phenotypic data, the targeted region containing *qSE7* was finally further mapped to a narrow region.
